# Prevalence of trigeminal neuralgia in Jincheng, China

**DOI:** 10.3389/fneur.2025.1616565

**Published:** 2025-10-13

**Authors:** Jianbo Shen, Ximeng Chen, Mimi Li, Jiabao Zhu

**Affiliations:** ^1^Department of Neurosurgery, Jincheng People's Hospital, Shanxi Medical University, Jincheng, China; ^2^Department of Neurosurgery, Yuncheng Central Hospital, Shanxi Medical University, Yuncheng, China

**Keywords:** trigeminal neuralgia, prevalence, epidemiology, China, neuropathic pain

## Abstract

**Objective:**

Trigeminal neuralgia (TN) is a debilitating neuropathic pain disorder that affects the trigeminal nerve, often causing intense facial pain. The prevalence and epidemiology of TN remain under-researched, particularly in China. This study aimed to estimate the lifetime prevalence of TN in Jincheng City, central China, and to provide an initial epidemiological insight into its occurrence within the Chinese population.

**Methods:**

A cross-sectional, descriptive epidemiological study was conducted from September to December 2024. A total of 1,350 individuals were surveyed using a 16-question screening questionnaire to assess symptoms of TN. TN diagnoses were based on the third edition of the International Classification of Headache Disorders (ICHD-3).

**Results:**

The final sample included 1,283 participants, with four diagnosed cases of TN, yielding a crude prevalence rate of 312 per 100,000 individuals (95% CI: 8–616 per 100,000). The mean age of the patients was 56.00 ± 9.59 years, and the female-to-male ratio was 3:1. The majority of patients (75%) had right-sided TN, with the maxillary and mandibular branches being most commonly affected. One case was classified as symptomatic TN due to multiple sclerosis, while the others were diagnosed with classical TN. All patients received pharmacological treatment, including carbamazepine and pregabalin.

**Conclusion:**

This study provides the first epidemiological data on TN prevalence in Jincheng City, China. The estimated prevalence aligns with findings from several international studies, though regional differences remain. The female predominance in TN cases and the higher incidence of right-sided involvement are consistent with global trends. Further studies with larger sample sizes and updated diagnostic criteria are needed to explore potential regional and genetic factors influencing TN prevalence and to assess the long-term impact of TN on public health in China.

## Introduction

Trigeminal neuralgia (TN) is a chronic neuropathic pain condition characterized by spontaneous, paroxysmal, electric shock-like or stabbing pain in specific regions of the face. It is a common cause of orofacial pain. The trigeminal nerve, a mixed nerve composed of special visceral motor fibers and general somatic sensory fibers, is responsible for sensory innervation of the face, mouth, and scalp, as well as motor control of the masticatory muscles. The trigeminal nerve is divided into three branches: the ophthalmic nerve (V1), the maxillary nerve (V2), and the mandibular nerve (V3) (1–3).

In clinical practice, TN commonly affects the teeth of the upper and lower jaws, with patients often initially seeking care from dental practitioners. Due to misdiagnosis or inappropriate treatment, irreversible dental procedures or extractions are frequently performed. As the condition progresses over time, the frequency and duration of TN episodes increases, severely impacting patients’ quality of life. In many cases, TN is associated with psychiatric comorbidities such as depression and anxiety (4–6), and in extreme cases, it may even lead to suicidal ideation (7).

The actual prevalence of TN is difficult to ascertain, as this condition is rare and not commonly studied in population-based research. Community-based studies on TN are limited, and prevalence rates range from 0.3 to 1,600 per 100,000 individuals (8–11). Additionally, research on the prevalence of idiopathic facial pain is sparse, with one study estimating the prevalence at 30 per 100,000 individuals (9). A prevalence study conducted in Turkey found the incidence of TN to be 52.1 per 100,000 individuals (12). Although some studies have been conducted on TN globally, the prevalence of TN in China remains largely unknown. Therefore, the present study aims to estimate the lifetime prevalence of TN in the central region of Jincheng city, China, contributing valuable data to the understanding of TN’s prevalence within the Chinese population.

## Methods

This is a cross-sectional descriptive epidemiological study. The research was conducted over a period of 4 months, from September 1, 2024, to December 31, 2024. The study was approved by the Ethics Committee of Jincheng People’s Hospital (approval number JCPH. No20210918001). The reasons for selecting Jincheng city for this study are several: it has a stable and homogeneous Chinese population, with an age and gender distribution similar to that of the general Chinese population; it is located in central China; and it has a well-organized city center with cooperative and helpful local government officials.

### Sample size

The sample size for estimating the prevalence of trigeminal neuralgia (TN) in the general population of Jincheng city, China, was calculated based on standard methods for cross-sectional surveys. The calculation was performed using a 95% confidence level (Z-value of 1.96), an expected prevalence rate of 0.3% (0.003) based on similar studies in other countries, and a margin of error of 5% (0.05). To account for potential clustering effects, a design effect (DE) of 0.8 was applied. Using the sample size formula, the required sample size was initially calculated as 1,277. After adjusting for the design effect (DE = 0.8), the sample size increased to 1,022. To account for possible non-responses or incomplete data, a 10% increase was applied, bringing the final sample size estimate to 1,125 participants. Therefore, the final sample size was set to 1,300 participants. This sample size is sufficient to estimate the lifetime prevalence of TN in Jincheng city with a 95% confidence level and a margin of error of 5%, ensuring reliable and meaningful results for the target population.

### Screening questionnaire

A 16-question questionnaire was used during the screening process. It included demographic information, symptoms of trigeminal neuralgia, imaging findings, and medication treatments. The questionnaire was based on a previously validated screening tool for trigeminal neuralgia [https://www.wjx.cn/xz/143817171.aspx]. This structured questionnaire was developed based on prior literature and expert consensus to assess TN. Content validity was established through review by three senior neurology specialists with more than 10 years of clinical experience. The questionnaire was pilot-tested in a small sample (*n* = 30) of patients to ensure clarity and feasibility. Internal consistency was acceptable, with a Cronbach’s *α* of 0.84, indicating good reliability.

### Screening team

Two 3-member teams were formed to identify potential patients and ensure reliable diagnoses of trigeminal neuralgia. Each team consisted of a neurologist, a nurse, and a medical student. All team members underwent a 7-day training program on trigeminal neuralgia screening, led by neurologists and public health experts.

### Study procedure

Preparatory work began one month prior to the screening to encourage full participation. Informational banners and billboards were used to explain the procedure. Announcements were made three times a week via loudspeakers to inform citizens. The selected households for screening were randomly chosen based on the population of neighborhoods and streets using a city map. Jincheng city is divided into 20 administrative streets; the number of households sampled from each street was proportional to its population size, ensuring representativeness. Within each street, households were selected by simple random sampling. Participants were aged 18 years or older. Screening visits were conducted by the teams every weekend from 8:30 a.m. to 5:00 p.m. Each household visited was provided with a questionnaire, and the questions were asked directly by the team members. Additionally, an electronic version of the questionnaire was made available online for further participation. The rationale for using an electronic version was to maximize participation among residents who were not available during face-to-face visits, thereby reducing non-response bias and improving overall coverage. All participants provided written informed consent. Individuals who answered affirmatively one or more of the questions related to trigeminal neuralgia symptoms during the visit were immediately examined on-site by the neurologist from the screening team. Participants with symptoms of trigeminal neuralgia were invited to undergo clinical examination and evaluation by senior neurologists. For patients who were unable to visit the hospital, arrangements were made for senior neurologists to conduct home visits.

### Diagnosis

Trigeminal neuralgia was diagnosed and classified according to the third edition of the International Classification of Headache Disorders (ICHD-3) (13). TN was defined by recurrent, paroxysmal, unilateral facial pain localized to one or more branches of the trigeminal nerve, without radiation beyond the affected area. The pain must meet the following characteristics: it lasts from a fraction of a second to two minutes, is of severe intensity, and is described as electric shock-like, shooting, stabbing, or sharp in quality. Furthermore, the pain is typically triggered by innocuous stimuli within the affected trigeminal distribution. Finally, the diagnosis of TN must not be better explained by another condition as defined in ICHD-3. The patients’ imaging findings were reviewed, and MRI scans were performed at a university hospital when necessary. Additionally, all medical records of patients diagnosed with trigeminal neuralgia were further examined.

### Statistical analysis

Data were recorded electronically and analyzed using SPSS 20.0 (version 20.0, SPSS Inc., Chicago, IL) to calculate frequency distributions and percentages. Statistical significance was set at *p* < 0.05.

## Results

A total of 1,350 individuals were surveyed. After excluding 66 individuals who declined to participate, 1,283 participants were ultimately included in this study. During the screening process, four participants were diagnosed with TN, yielding a crude prevalence rate of 312 per 100,000 individuals (95% CI: 8–616 per 100,000). The mean age of TN patients was 56.00 ± 9.59 years (range: 43–65 years), with a female-to-male ratio of 3:1. One patient with multiple sclerosis exhibited pontine plaques on MRI without evidence of neurovascular compression, leading to a classification of symptomatic TN. The remaining three patients presented with unilateral trigeminal nerve atrophy, and MRI findings confirmed vascular compression, consistent with a diagnosis of classical TN.

Three patients (75%) had right-sided involvement, while one patient (25%) had left-sided involvement. Among them, one patient exhibited isolated maxillary nerve involvement, another had isolated mandibular nerve involvement, and two patients demonstrated concurrent involvement of both the maxillary and mandibular branches.

All patients received monotherapy or combination therapy with antiepileptic drugs, including carbamazepine, oxcarbazepine, pregabalin, and gabapentin. None of the patients reported a history of chronic conditions such as diabetes or hypertension. Table 1 summarizes the demographic characteristics of TN patients, while Table 2 presents the clinical features of the four diagnosed cases.

**Table 1:** Demographic and clinical characteristics of participants.

Number of participants	1,283
Participation rate	95.03%
Number of cases	4
Crude prevalence	312/100.000
Mean age of all participants (years)	50.64 ± 10.2
Female/male ratio	3/1
Age of patients (years)	56.00 ± 9.59
Classical/Secondary TN	3/1
Right/left involvement	3/1
Affected branches	
Opthalmic (V1), *n* (%)	–(0%)
Maxillary (V2), *n* (%)	1 (25%)
Mandibular (V3), *n* (%)	1 (25%)
Maxillary+Mandibular, *n* (%)	2 (50%)

TN, trigeminal neuralgia.

**Table 2:** Characteristics of trigeminal neuralgia patients.

Trigeminal neuralgia patients	Patients No.			
	1	2	3	4
Age (years)	65	55	43	61
Gender	Female	Male	Female	Female
Pain location	V2	V2 + V3	V2 + V3	V3
Pain days per month	10	2	7	3
Pain intensity (VAS)	9	8	7	8
Pain triggers	Eat	Cold	Touch	Eat
Adequate treatment	CBZ	CBZ	CBZ	CBZ/OXC
Vessel-nerve contact on MRI	−	+	+	+

Visual analog scale; V1, V2, and V3, first, second, and third branch of the trigeminal nerve, respectively; CBZ, carbamazepine; OXC, oxcarbazepine.

## Discussion

This study provides the first epidemiological data on TN in the central region of China, specifically in Jincheng City. The estimated prevalence of TN in our study population was 312 per 100,000 individuals (95% CI: 8–616 per 100,000), which falls within the range reported in prior international studies but exhibits some regional variation. Consistent with previous findings, TN was more frequently observed in females, with a female-to-male ratio of 3:1. Additionally, right-sided involvement was predominant, occurring in 75% of cases. The maxillary and mandibular branches were most commonly affected, either in isolation or combination, while no cases of ophthalmic nerve involvement were identified.

The prevalence estimates for TN vary considerably across studies, likely due to differences in study design, sample size, diagnostic criteria, and population characteristics. Globally, previous systematic reviews have reported the prevalence of TN ranged from 30 per 100,000 (95% CI: 10–80) to 300 per 100,000 (95% CI: 160–550) (14). Regionally, a large-scale UK study reported a lifetime prevalence of 70 per 100,000 (8), while a Norwegian face-to-face survey found a prevalence of 108.8 per 100,000 among adults aged 18 years and older (11). Notably, an older Italian cohort study reported an exceptionally high prevalence of 1,600 per 100,000 in an elderly population (aged 55–94 years), highlighting the impact of age distribution on prevalence estimates (10). In contrast, a recent Egyptian study reported a lower prevalence of 29.5 per 100,000 in individuals over 37 years old (15). Recent study report TN prevalence in Turkey, revealed a relatively low crude rate compared to international data (52.1/100,000) (12). Our findings are more aligned with studies conducted in Norway and France but differ from those in Egypt, Turkey and Italy, suggesting possible genetic, environmental, and methodological influences on TN prevalence. These discrepancies may also reflect heterogeneity in study methodology, such as differences in case ascertainment (self-reported surveys vs. clinical diagnosis), diagnostic criteria applied, age composition of the studied populations, and sample size variability. Generally, the extremely high prevalence in the Italian elderly cohort is likely explained by the advanced age of participants, while lower prevalence in Egypt and Turkey may be related to narrower age ranges and possible under-reporting of symptoms.

Right-sided TN involvement has been consistently reported as more common in epidemiological studies, with reported rates ranging from 56 to 75% (9, 11, 16–18). In our study, three out of four TN patients (75%) had right-sided involvement, supporting this trend. While the underlying cause of this asymmetry remains unclear, some studies have suggested a potential association with vascular anatomy and asymmetrical neurovascular compression.

Consistent with previous epidemiological data, our study further supports the marked sex difference in the prevalence of TN. In our sample, 75% of patients diagnosed with TN were female, in contrast to the majority of prior studies which reported a higher prevalence among females, often with female-to-male ratios ranging from 1.5:1 to 4:1 (12, 19). Previous systematic review has also documented the proportion between women and men who had TN was 3 to 1 (14). This discrepancy in our findings may be attributed to the small sample size or regional demographic factors, but it also underscores the need for further large-scale studies to clarify potential sex-related biological or environmental influences in TN pathophysiology.

Despite providing valuable epidemiological insights, this study has several limitations. First, the relatively small number of TN cases limits the precision of our prevalence estimate and subgroup analyses. Additionally, potential recall bias may have influenced symptom reporting, and the sample may not fully represent the broader population. Second, given the statistical nature of this work, a more rigorous statistical review may help to refine our estimates and provide additional validation of our findings. Third, while we utilized standardized diagnostic criteria, the evolving classification of TN suggests that future studies should consider incorporating updated diagnostic frameworks. Finally, we acknowledge that epidemiological data from a single city represent a narrow geographic scope and may have limited generalizability. Nevertheless, such region-specific data remain valuable in highlighting local disease burden, informing healthcare planning, and contributing to global comparative data on TN prevalence.

## Conclusion

This study contributes novel epidemiological data on TN prevalence in China, with findings that are comparable to several international studies. While TN prevalence in our study falls within previously reported ranges, differences in study methodologies and population characteristics may account for global variations. The association between TN and MS observed in our study warrants further investigation through larger-scale epidemiological research. Future studies should also explore potential risk factors and regional influences on TN prevalence to better inform clinical and public health strategies.

## Data Availability

The original contributions presented in the study are included in the article/supplementary material, further inquiries can be directed to the corresponding author/s.
